# Real-time flow assessment: From model to patients

**DOI:** 10.1186/1532-429X-16-S1-P386

**Published:** 2014-01-16

**Authors:** Julius Traber, Lennart Wurche, Matthias A Dieringer, Wolfgang Utz, Andreas Greiser, Ning Jin, Philipp Barckow, Jeanette Schulz-Menger

**Affiliations:** 1 Working Group on Cardiovascular Magnetic Resonance Imaging of Experimental and Clinical Research Center/Department of Cardiology and Nephrology, Charité Campus Buch/HELIOS Klinikum Berlin-Buch, Berlin, Germany; 2 Healthcare Sector, Siemens AG, Erlangen, Germany; 3 Medical Solutions, Siemens USA Inc., Columbus, Ohio, USA; 4 Circle Cardiovascular Imaging Inc., Calgary, Alberta, Canada

## Background

In stratification of heart valve diseases blood flow assessment often plays a key role. When echocardiography struggles, phase contrast magnetic resonance imaging (PC-MRI) may be considered as an alternative (Srichai et al. AJR 2009). Arrhythmias are a major limitation of conventional segmented PC-MRI (SEG). Real-time sequences (RT) could overcome it. The purpose of this study is to evaluate RT in a flow model as well as in volunteers and patients. We hypothesize to measure equal velocities and flow compared to SEG as reference in sinus rhythm and aimed to show feasibility in atrial fibrillation (Afib).

## Methods

**Figure 1:**
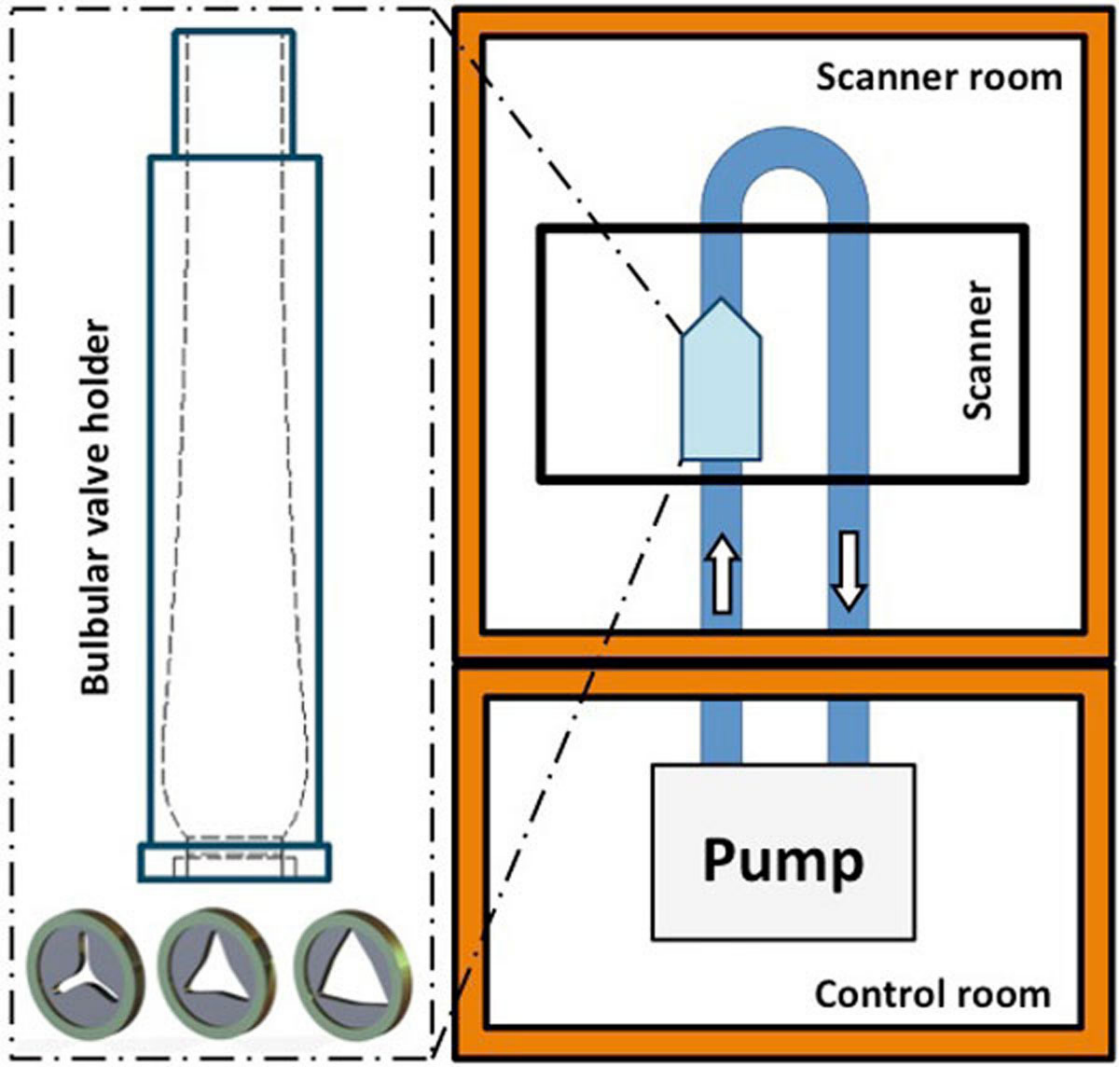
**Flow model set-up: Adjustable pump generates constant flow; measurements 25 mm behind interchangable aortic stenosis-like narrowings**.

In a flow model (I), volunteers and prospectively enrolled patients (II) we compared a highly accelerated RT (temp. res. 40 ms, TE 5.6 ms, ETL 7, T-PAT 3×, matrix 128×104px) using shared velocity encoding (Lin et al. MRM 2009) with SEG (temp. res. 48 ms, TE 2.3 ms, ETL 5, I-PAT 2×, matrix 192×156px) on a 1.5 T scanner (Avanto, Siemens Healthcare, Germany) with a 12 channel cardiac coil. I The model generated adjustable constant flow. 81 PC images were acquired 25 mm from an interchangeable aortic stenosis-like narrowing with different areas (0.6 cm2, 1.3 cm2, 2.0 cm2) perpendicular to tube running (Figure 1). II In vivo studies were measured at sinotubular junction perpendicular to the aorta. We quantified (cvi42, Circle CVI, Canada) with equalized voxel size: in I mean velocity and flow in II mean peak velocity, stroke volume and regurgitation fraction In patients with Afib only RT was applied.

## Results

**Figure 2:**
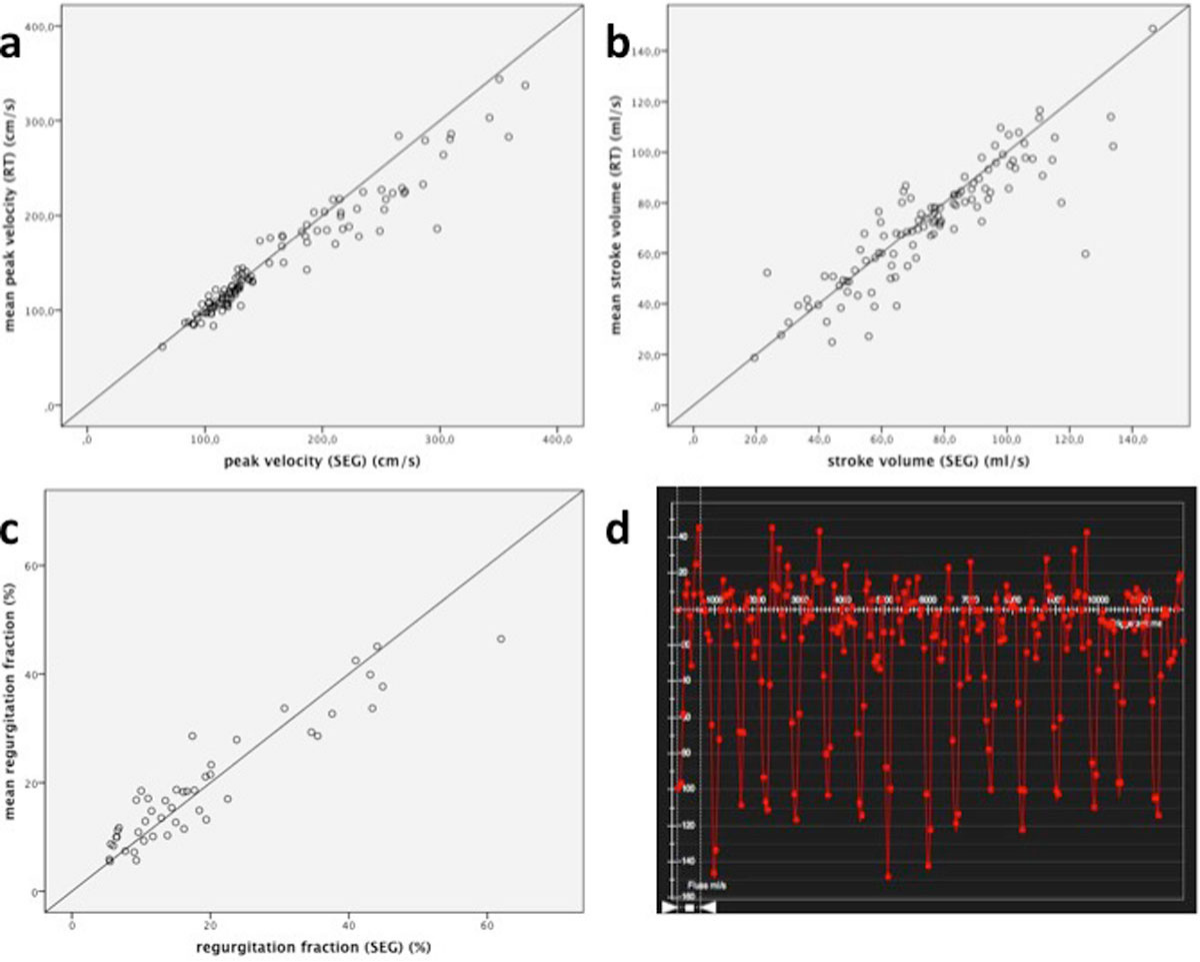
**In vivo results: RT shows good correlation to SEG regarding peak velocity (a), stroke volume (b) and regurgitation fraction (c)**. Stray bullets had at least moderate aortic valve stenosis. On Afib RT shows frequency-dependent variability of stroke volumes (d).

I All PC images were acquired successfully and evaluable. Mean velocities ranged 35-216 cm/s (SEG). Scatter plots showed good correlations between SEG and RT (velocity: r = 0.991, p < 0.0005; flow: r > 0.993, p < 0.0005). Flow in RT partially strayed to higher values, although differences were not significant (122 ± 72 ml/s vs. 143 ± 74 ml/s; p = 0.290). II We included 119 subjects: 52 healthy subjects (28 men, 51 ± 19 y) as well as patients (55 men, 66 ± 15 y) with aortic valve disease (60) and/or Afib (8). RT acquisition failed in one, image quality was non-diagnostic in three cases. Peak velocities ranged 64-373 cm/s (SEG). Scatter plots showed reasonable correlations between SEG and RT (velocity: r = 0.964, p < 0.0005; stroke volume r = 0.880, p < 0.0005). Velocities in RT partially strayed to lower values on high reference velocities, although differences were not significant (164 ± 71 cm/s vs. 153 ± 60 cm/s; p = 0.206). Stray bullets had at least moderate aortic valve stenosis. In patients with aortic valve insufficiency (47), regurgitation fractions correlated well (r = 0,937; p = 0,0005). In Afib patients PC-RT was feasible in all patients and flow-time plots showed frequency-dependent variability of stroke volumes (Figure 2).

## Conclusions

The evaluated real-time PC sequence can access flow reliably and in good correlation to a conventional segmented version in model experiments, volunteers and patients. Hence it might become an useful alternative to doppler echocardiography in arrhythmic patients.

## Funding

Funded by the general research budget of the working group.

